# Long noncoding RNA MALAT1 promotes cell proliferation through suppressing miR-205 and promoting SMAD4 expression in osteosarcoma

**DOI:** 10.18632/oncotarget.20678

**Published:** 2017-09-06

**Authors:** Qingbo Li, Xiaohan Pan, Xiqian Wang, Xiejia Jiao, Jiachun Zheng, Zhiqiang Li, Yanqing Huo

**Affiliations:** ^1^ Department of Orthopedics, The Second Hospital of Shandong University, Jinan, 250133, Shandong Province, China; ^2^ Department of Health Management, The Second Hospital of Shandong University, Jinan, 250133, Shandong Province, China

**Keywords:** MALAT1, osteosarcoma, miR-205, proliferation, SMAD4

## Abstract

Increasing evidences have indicated that long non-coding RNAs (lncRNAs) play an important role in multiply biological processes including cell development, differentiation, proliferation and invasion. The metastasis-associated lung adenocarcinoma transcript 1 (MALAT1), is a highly conserved nuclear ncRNA and a key regulator of metastasis development in several cancers. However, its role in osteosarcoma progression is not well known. In this study, we sought to determine the clinical and bilogical role of MALAT1 in osteosarcoma progression. RT-qPCR analysis showed that MALAT1 expression was significantly increased in primary osteosarcoma tissues and cell lines. Kaplan-Meier analysis indicated that patients with high expression of MALAT1 was associated with poor overall survival compared with the low expressing patients. Furthermore, the gain and loss function assay showed that miR-205 was suppressed by MALAT1 in osteosarcoma and this interaction between miR-205 and MALAT1 has reciprocal effects. Cell viability assay showed that MALAT1 promoted MG-63 and SAOS-2 cell growth through suppressing miR-205. Subsequently, the downstream gene SMAD4 was identified as a direct functional target of miR-205, and miR-205 suppressed osteosarcoma cell growth through suppressing SMAD4. Finally, we demonstrated that MALAT1 promoted osteosarcoma progression via a miR-205-SMAD4 axis. In conclusion, we revealed that enhanced MALAT1 expression predicted unfavourable outcome in osteosarcoma and promoted cell proliferation through suppressing miR-205 and activating SMAD4 function. Thus, lncRNA MALAT1 may serve as a promising prognostic and therapeutic target for osteosarcoma patients.

## INTRODUCTION

Osteosarcoma is the most common kind of primary bone tumors with high morbidity in infants and adolescents [1]. Though great effort have been exerted to investigate the underlying mechanism of OS progression during the past decades, the prognosis of osteosarcoma remains poor. Currently, most osteosarcoma patients died for the distant metastasis, especially in pulmonary [2]. The primary treatment for osteosarcoma is a combination of surgery and chemotherapy. However, osteosarcoma frequently develops resistance to conventional chemotherapy regimens. Further exploration of this area will help in the development of effective strategies in the diagnosis, treatment and prognosis of osteosarcoma.

Long noncoding RNAs (lncRNAs) are most commonly defined as RNA transcript of more than 200 nucleotides (nt) and located in nuclear or cytosolic fractions with no protein-coding capacity [3]. Recent studies have discovered that long non-coding RNAs (lncRNAs) play an important role in multiply biological processes including cell development, differentiation, proliferation, invasion, and migration [4–7]. The metastasis-associated lung adenocarcinoma transcript 1 (MALAT1), is located on the chromosome 11q13 and was firstly found as a predictive biomarker for metastasis in the early stage of non-small cell lung cancer [8]. More recently, MALAT1 was identified to have diverse effect on carcinogenesis, such as cell proliferation and apoptosis in colorectal cancer, gastric cancer and pancreatic cancer. However, its role in osteosarcoma has not been identified.

Over the last decade, microRNAs (miRNAs) have emerged as key players in carcinogenesis. Aberrant expression of miRNAs has been demonstrated to play a critical role in the initiation and progression of several cancers [9]. MiRNAs regulate gene expression primarily via their interaction with the 3’UTRs of target mRNAs, resulting in mRNA decay or translational repression [10]. In cancer, miRNAs can behave as oncogenes or tumor suppressor genes depending on the cellular function of their target [11]. It is reported that miR-205 may act as an antioncogene in several cancer types including breast cancer, prostate cancer and osteosarcoma [12]. The crosstalk between lncRNAs and microRNAs (miRNAs) has recently been reported to contribute to the pathogenesis of diseases, including cancer [13]. Some lncRNAs play important roles in the regulation of gene expression by acting as competing endogenous RNAs [14]. In addition, further investigation shows that lncRNAs are more effective ceRNAs without any interference with translation [15]. However, the interaction between lncRNA and miRNAs in the osteosarcoma is not well known and needs further investigation.

In our current study, we sought to identify the regulatory role of MALAT1 in osteosarcoma cell proliferation, and further identify the direct target correlated with the malignant phenotype of osteosarcom. The results indicated that MALAT1 was upregulated while miR-205 was suppressed in osteosarcoma. SMAD4 was identified as a functional target gene of miR-205 in osteosarcoma. More importantly, MALAT1 promoted osteosarcoma cell proliferation through suppressing miR-205 and activating SMAD4 signaling.

## RESULTS

### MALAT1 was up-regulated and related to poor survival in osteosarcoma patients

**Figure 1:**
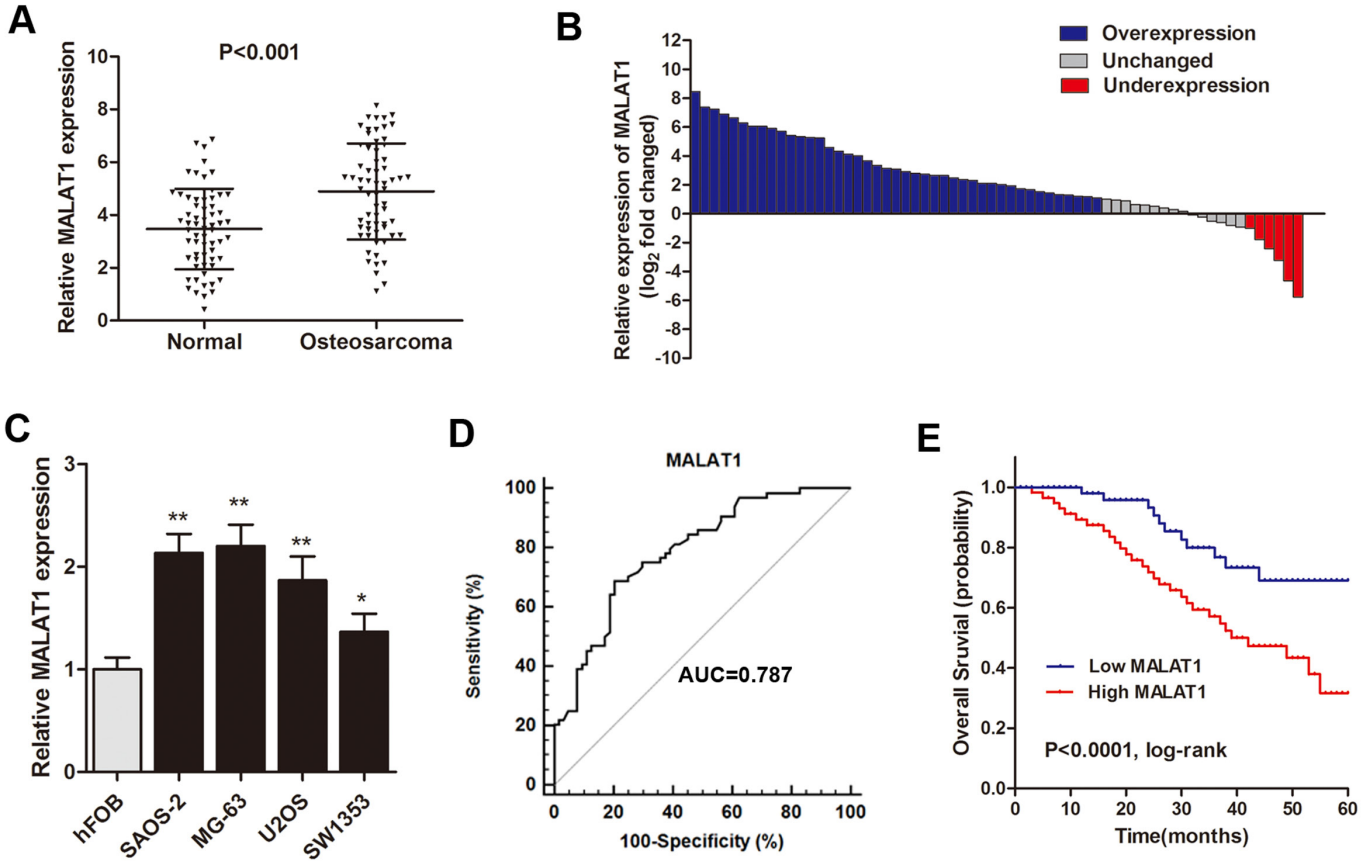
MALAT1 was up-regulated and related to poor survival in osteosarcoma patients **(A)** RT-qPCR showed that MALAT1 was significantly up-regulated in primary osteosarcoma tissues compared to noncancerous tissues; **(B)** The MALAT1 expression level was analyzed using RT-qPCR and expressed as log_2_ fold change (CRC/normal), and the log_2_ fold changes were presented as follows: >1, overexpression (43 cases); <1, underexpression (6 cases); the remainder were defined as unchanged (15 cases); **(C)** MALAT1 was significantly up-regulated in four osteosarcoma cell lines compared with normal osteoblastic cells; **(D)** ROC curve analysis was performed to investigate the diagnostic value of MALAT1 level in discriminating osteosarcoma patients from healthy individuals; **(E)** Kaplan-Meier curves for overall survival according to the MALAT1 levels from the primary tissues of osteosarcoma patients. ^*^P<0.05, ^**^P<0.01.

RT-qPCR was used to detect MALAT1 expression in 64 primary osteosarcoma tissues and paired adjacent noncancerous tissues, normalized to GAPDH. Our results showed that MALAT1 was up-regulated in primary osteosarcoma tissues compared to noncancerous tissues (P<0.001, Figure 1A). Additionally, the osteosarcoma tissues in 66.2% (43 of 64) of cases had at least 2-fold higher expression of MALAT1 than noncancerous tissues (Figure 1B). Subsequently, the MALAT1 expression in four osteosarcoma cell lines (MG-63, SAOS-2, U2OS, SW1353) and one osteoblastic cell line (hFOB) were also determined. As shown in Figure 1C, MALAT1 was up-regulated in all the four osteosarcoma cell lines compared with normal osteoblastic cells. We then analyzed the association between MALAT1 expression with clincopathological factors among the 64 osteosarcoma patients. High MALAT1 level was significantly correlated with enhanced tumor size, pulmonary metastasis and TNM stage, but not correlated with other factors such as gender, age and differentiation (Table 1).

**Table 1:** Association between MALAT1 expression and clinical parameters in 64 osteosarcoma patients [median (interquartile range)]

Characteristics	Number	MALAT1 expression	P-value
Gender			0.735
Male	39	4.56 (0.90-7.69)	
Female	25	4.85 (1.57-8.12)	
Age (years)			0.415
<40	33	4.91 (1.20-8.26)	
≥40	31	4.62 (1.48-8.53)	
Tumor size			0.012
<6cm	34	3.72 (1.20-8.18)	
≥6cm	30	5.19 (1.57-7.96)	
Differentiation			0.193
Well	23	4.66 (1.20-7.61)	
Moderate	30	4.86 (1.48-7.97)	
Poor	11	5.09 (1.35-8.53)	
Pulmonary metastasis			0.000
Yes	22	3.27 (1.20-7.11)	
No	42	6.39 (2.99-8.53)	
TNM stage			
I-II	29	4.04 (1.20-7.64)	0.018
III-IV	35	5.99 (1.87-8.53)	

Subsequently, Receiver operating characteristic (ROC) analysis was used to evaluate the diagnostic performance of MALAT1. The area under the ROC curve (AUC) for MALAT1 was 0.787 (95% confidence interval [CI] = 0.706–0854) and the optimal cut-off value was 4.94, providing a sensitivity of 68.7 % and a specificity of 79.7 % (Figure 1D). We then divided the patients into a high and a low expressing group by using the median value (4.77) of 64 samples, and the Kaplan-Meier analysis indicated that patients with high expression of MALAT1 was associated with poor overall survival rate compared with the low expressing patients (Figure 1E). Moreover, Cox regression mutivariate analysis showed that high MALAT1 was significantly associated with poor survival prognosis independent with other clinical covariates (Table 2). Collectively, these results suggest that MALAT1 is up-regulated and could be an independent prognostic factor in osteosarcoma.

**Table 2:** Univariate and multivariate cox proportional hazards regression model analysis for overall survival in osteosarcoma patients

Characteristics	Univariate analysis			Multivariate analysis		
	HR	95% CI	*P* value	HR	95% CI	*P* value
Gender	0.999	0.546-2.648	0.699			
Age	1.215	0.663-3.384	0.227			
Tumor size	1.743	0.639-3.520	0.357			
Differentiation	1.724	0.701-3.392	0.224			
TNM stage	2.338	1.162-3.891	0.033	2.349	1.287-4.367	0.053
Pulmonary metastasis	3.714	1.646-7.831	0.010	3.679	1.502-7.792	0.005
MALAT1 expression	3.325	1.430-4.908	0.015	3.452	1.320-5.474	0.011

### MALAT1 suppressed miR-205 expression in osteosarcoma cells

**Figure 2:**
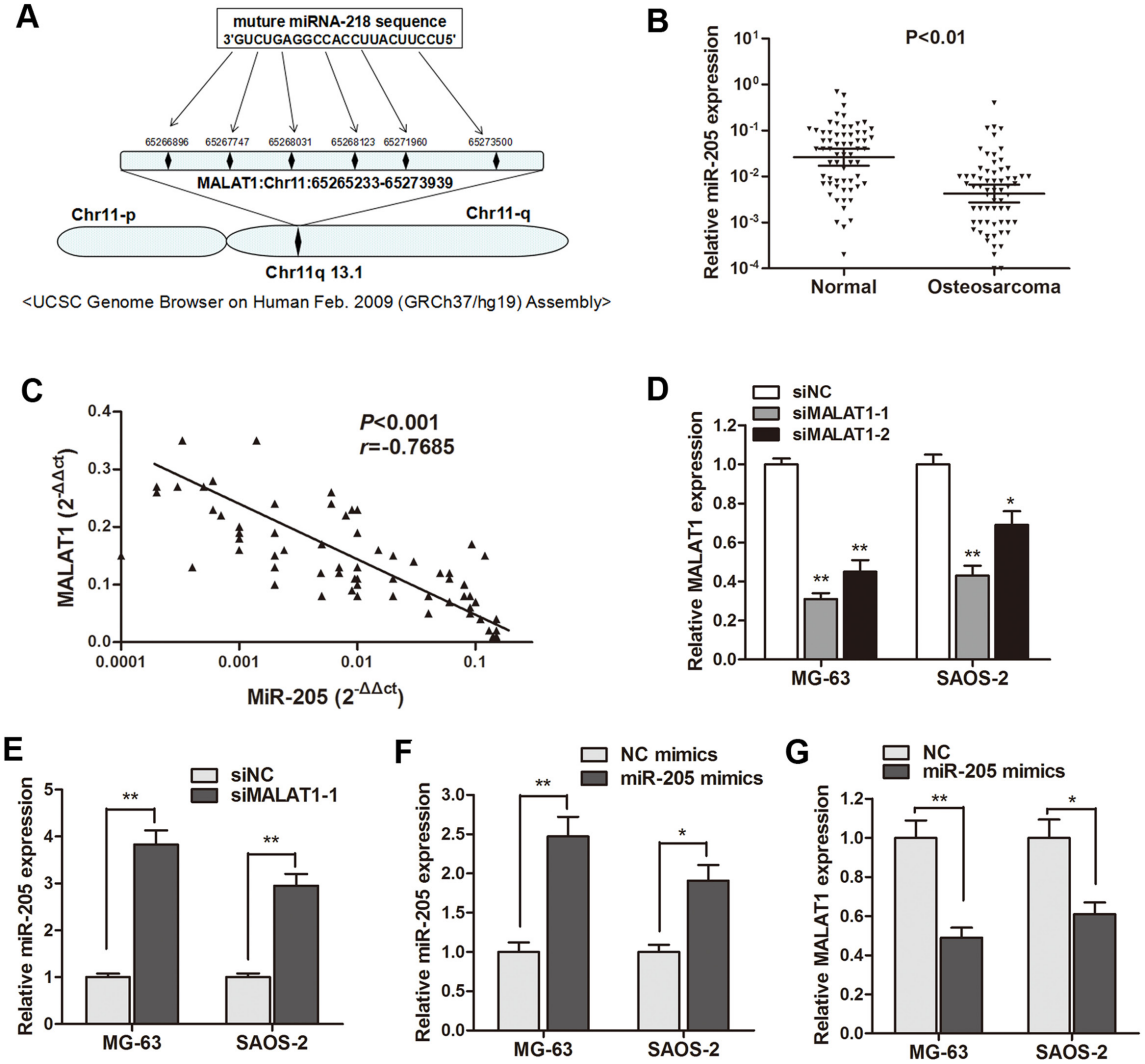
MiR-205 was negatively regulated by MALAT1 in osteosarcoma **(A)** Representation of the miR-205 binding site in MALAT1 based on miRcode (http://www.mircode.org/mircode/); **(B)** RT-qPCR results showed that miR-205 expression level was significantly down-regulated in primary osteosarcoma tissues compared with noncancerous tissues; **(C)** A significant negative correlation was found between the miR-205 levels and the MALAT levels in 64 primary osteosarcoma tissues by Spearman testing; **(D)** MALAT1 was silenced by both si-MALAT1-1 and si-MALAT1-2 in MG-63 and SAOS-2 cells; **(E)** MiR-205 was significantly up-regulated in MG-63 and SAOS-2 cells transfected with si-MALAT1-1 when compared with cell transfected with si-NC; **(F)** The transfection of miR-205 mimics significantly promoted miR-205 expression in both osteosarcoma cells. **(G)** MALAT1 expression was down-regulated by transfection of miR-205 mimics in MG-63 and SAOS-2 cells. ^*^P<0.05, ^**^P<0.01.

Based on the above observations, we sought to define the underlying mechanism that may account for the clinical findings. Based on the bioinformatics analysis (http://www.mircode.org/mircode) [16], we identified six miR-205 binding sites on the MALAT1 sequence (Figure 2A). As miR-205 has been reported to be involved in osteosarcoma progression [12], we focus on the interaction between MALAT1 and miR-205. While the expression of MALAT1 was higher in osteosarcoma tissues and cell lines, miR-205 expression was significantly lower (P<0.01, Figure 2B). Additionally, a significant negative correlation was also found between MALAT1 and miR-205 expression in 64 primary osteosarcoma tissues (Figure 2C). As shown in Figure 2D, MALAT1 was significantly silenced by siMALAT1-1 and siMALAT1-2. Subsequently, The gain and loss functional assay indicated that miR-205 was significantly increased after knockdown of MALAT1 in osteosarcoma cells (Figure 2E), while enhanced expression of miR-205 dramatically silenced MALAT1 expression level in osteosarcoma cells (Figure 2F and 2G). These data revealed that MALAT1 suppressed miR-205 expression in osteosarcoma, and the negative interaction between miR-205 and lncRNA MALAT1 has reciprocal effects.

### MALAT1 promoted cell proliferation through suppressing miR-205 level in osteosarcoma

**Figure 3:**
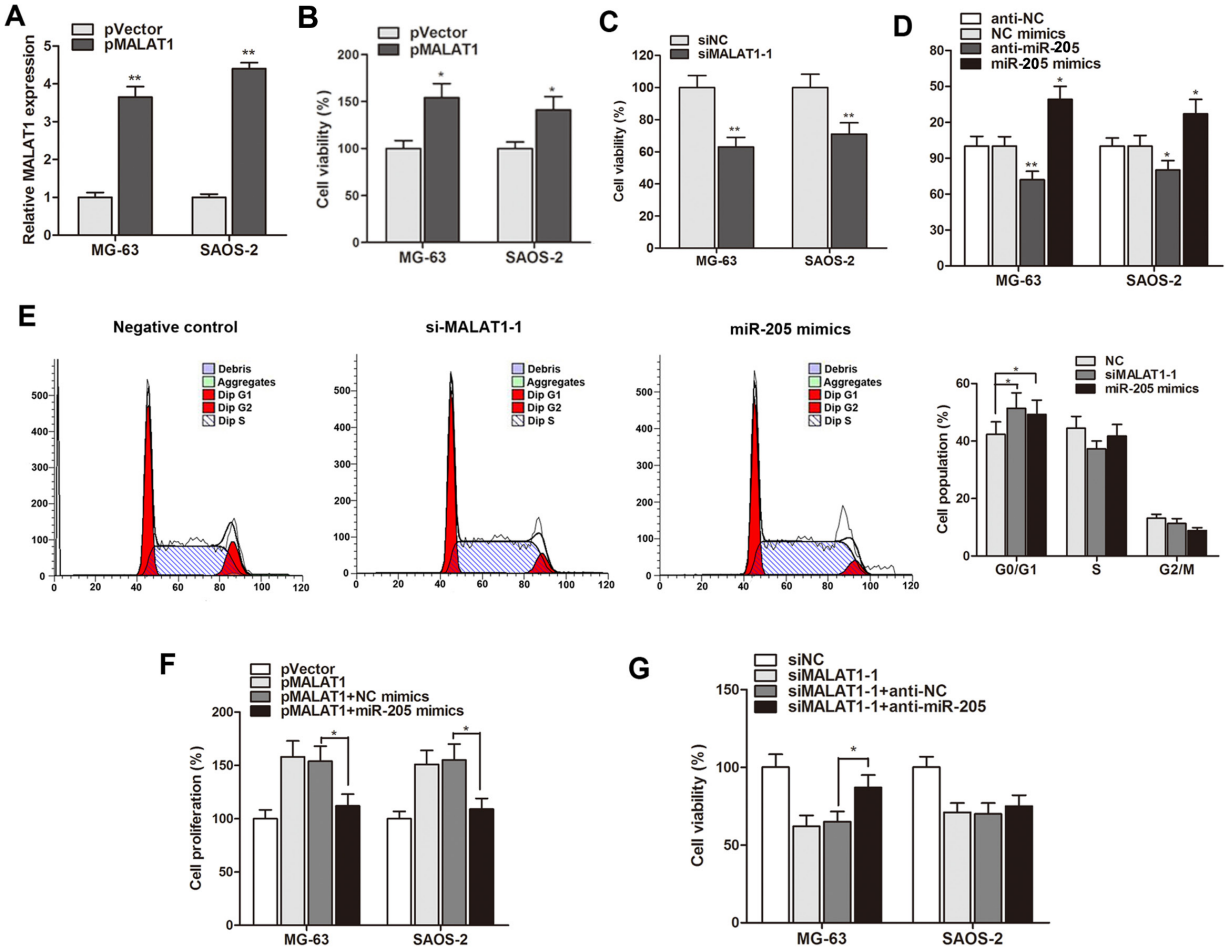
MALAT1 mediated cell proliferation and cell-cycle arrest through suppressing miR-205 expression **(A)** MALAT1 was dramatically up-regulated in osteosarcoma cells by the transfection of pMALAT1; **(B)** CCK8 assay showed taht pMALAT1 significantly promoted cell proliferation rate of both osteosarcoma cells; **(C)** Cell viability was significantly suppressed by siMALAT1-1; **(D)** MiR-205 induced a suppressive effect on cell proliferation whereas anti-miR-205 promoted cell proliferation rate in MG-63 and SAOS-2 cells; **(E)** Cell cycle analysis showed that siMALAT1-1 or miR-205 induced an increased percentage of MG-63 cells in G1/G0 phase compared with negative control; **(F-G)** The gain and loss function assay indicated that miR-205 mimics restrained the enhanced cell growth induced by pMALAT1, however, suppression of miR-205 significantly rescued the growth inhibition induced by siMALAT1-1 in MG-63 cells. ^*^P<0.05, ^**^P<0.01.

After having validated the dysregulation of MALAT1 and miR-205 in osteosarcoma, We then investigated their regulatory roles during osteosarcoma progression, and MALAT1 was significantly up-regulated by pMALAT1 and overexpression of MALAT1 dramatically promoted cell viability (Figure 3A and 3B), while siMALAT1-1 suppressed cell proliferation (Figure 3C). For miR-205, we found that miR-205 suppressed whereas anti-miR-205 promoted cell viability (Figure 3D). Take a step further, we examined the effect of MALAT1 or miR-205 on cell cycle phase. Both MALAT1 knockdown and miR-205 overexpression promoted the cell percentage in G1/G0 phase (Figure 3E).

In addition, we also investigated the antagonistic effect of MALAT1 and miR-205 on cell growth. As shown in Figure 3F, miR-205 restrained the enhanced cell growth induced by pMALAT1 in both MG-63 and SAOS-2 cells. In addition, suppression of miR-205 significantly rescued the growth inhibition induced by MALAT1 knockdown in MG-63 cells (Figure 3G).

### miR-205 inhibited the expression of SMAD4 via binding to its 3′UTR

**Figure 4:**
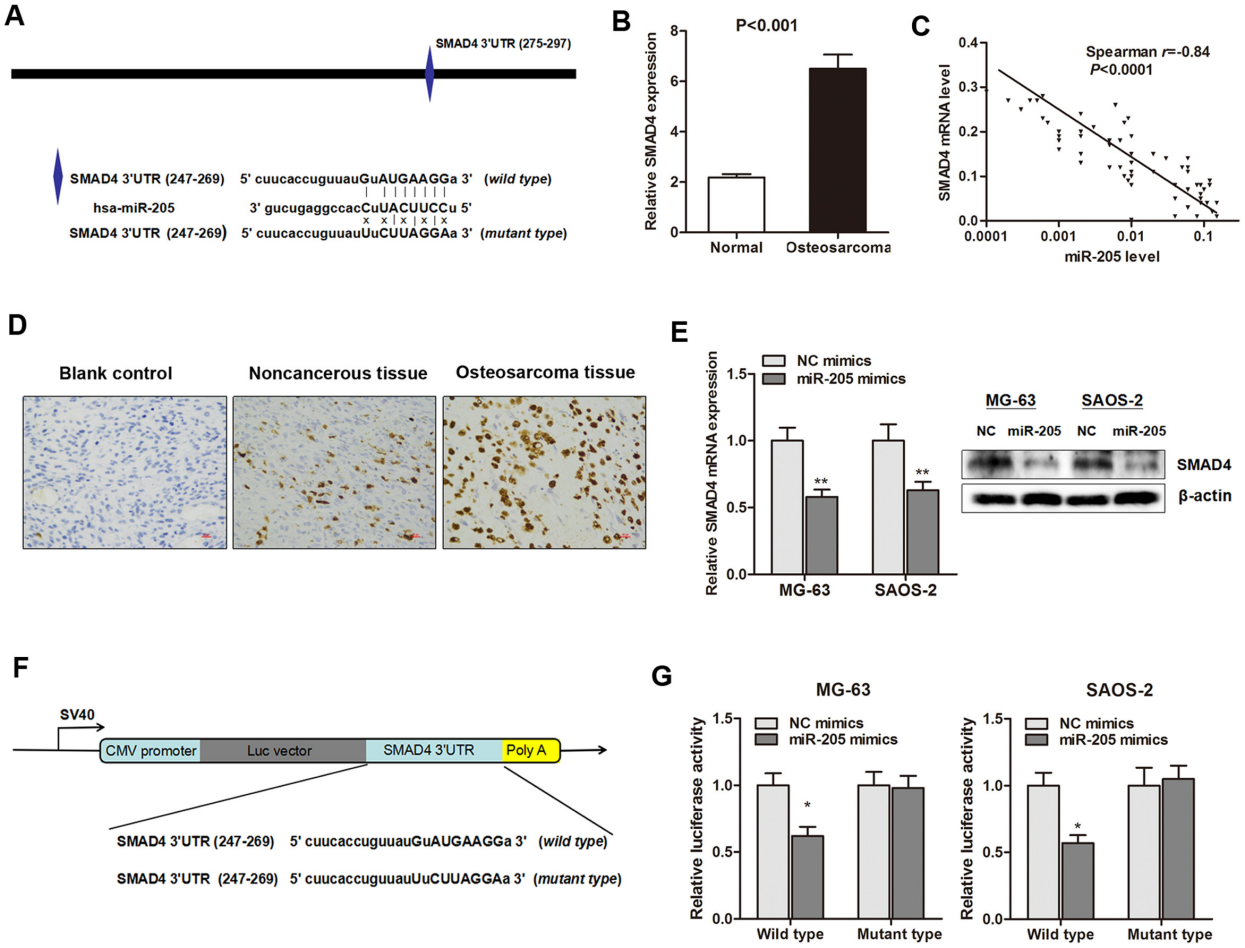
SMAD4 is a direct downstream target of miR-205 in osteosarcoma **(A)** Illustration of the putative predicted miR-205 binding site in the SMAD4 mRNA 3’UTR region, and the mutation within the binding site was generated; **(B)** SMAD4 mRNA expression level was significantly increased in primary osteosarcoma tissues when compared with normal tissues; **(C)** Spearman correlation test showed that miR-205 level was negatively correlated with SMAD4 mRNA level in the osteosarocma tissues; **(D)** Immunohistochemistry analysis indicated that SMAD4 protein was significantly enriched in primary osteosarcoma when compared with noncancerous tissues; **(E)** Overexpression of miR-205 significantly suppressed SMAD4 mRNA (left panel) and protein (right panel) expression level in both MG-63 and SAOS-2 cells; **(F)** The conserved miR-205 binding sequence or its mutated form was inserted into the C-terminal of the luciferase gene to generate pMIR-SMAD4-wild-3′UTR or pMIR-SMAD4-mut-3′UTR, respectively; **(G)** Dual-luciferase reporter assay indicates that miR-205 targets the wild-type but not the mutant 3′UTR of SMAD4. ^*^P<0.05, ^**^P<0.01.

As miRNAs function mainly through the inhibition of target genes, the targets of miR-205 that function in osteosarcoma cells were further investigated. The targets of miR-205 were predicted through at least three databases (Pictarget, miRnada and TargetScan) (Figure 4A), and SMAD4 was selected as a putative target, since it was reported to be involved in cancer proliferation [17]. RT-qPCR showed that SMAD4 mRNA was up-regulated in primary osteosarcoma tissues (Figure 4B), and a significant negative correlation was also found between SMAD4 and miR-205 expression in 64 osteosarcoma tissues (r=-0.85, *P*<0.0001, Figure 4C). Additionally, immunohistochemistry analysis indicated that SMAD4 protein was significantly enriched in primary osteosarcoma when compared with noncancerous tissues (Figure 4D). More importantly, endogenous SMAD4 mRNA and protein levels were markedly decreased after overexpression of miR-205 in MG-63 and SAOS-2 cells (Figure 4E).

To further identify whether miR-205 directly targets the 3’-UTR region of SMAD4 mRNA, we constructed a reporter vector consisting of the luciferase coding sequence followed by the 3’-UTR of SMAD4 (Figure 4F). We performed luciferase reporter assay with a vector containing the putative SMAD4 3’-UTR target site downstream of the luciferase reporter gene, which was transfected into osteosarcoma cells. Cotransfection of miR-205 significantly suppressed the luciferase activity of the reporter containing wild-type 3’-UTR but did not suppress the mutant reporter (Figure 4G). These data reveal that SMAD4 is a direct functional target of miR-205 and miR-205 inhibited the expression of SMAD4 via binding to its 3’UTR region.

### SMAD4 participates in miR-205-induced inhibition of cell growth in osteosarcoma

**Figure 5:**
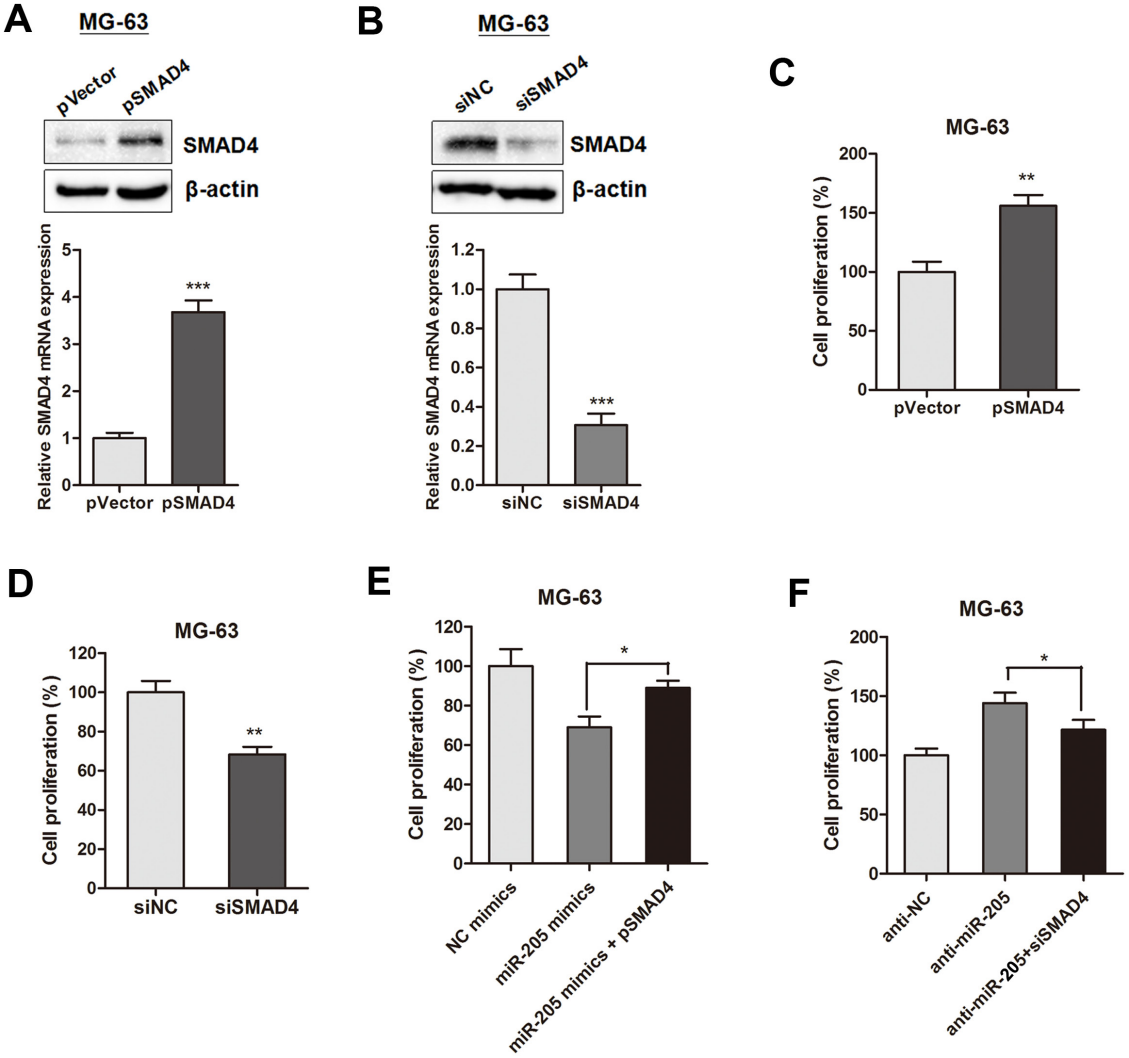
SMAD4 is involved in miR-205-induced inhibition of proliferation of osteosarcoma cells **(A-B)** SMAD4 expression was over-expressed or silenced by transfection with pSMAD4 (A) or siSMAD4 (B) in MG-63 cells, respectively; **(C-D)** CCK-8 assay indicated that pSMAD4 (C) promoted while siSMAD4 (D) suppressed MG-63 cell growth; **(E-F)** The gain and loss function assay showed that overexpression of SMAD4 partially reversed the miR-205 induced suppressive effect on osteosarcoma cell proliferation; while silence of SMAD4 restrained the enhanced cell viability induced by anti-miR-205. ^*^P<0.05, ^**^P<0.01, ^***^P<0.001.

Based on the above results, we tested if SMAD4 is responsible for the miR-205 induced tumorigenesis in osteosarcoma cells. SMAD4 expression was over-expressed or silenced by transfection with pSMAD4 or siSMAD4 in MG-63 cells, respectively (Figure 5A and 5B). CCK-8 assay indicated that pSMAD4 promoted while siSMAD4 suppressed MG-63 cell growth (Figure 5C and 5D). Moreover, gain and loss function assay showed that overexpression of SMAD4 partially reversed the miR-205-induced suppressive effect on cell proliferation (Figure 5E); while SMAD4 silencing restrained the enhanced cell viability induced by anti-miR-205 (Figure 5F).

### MALAT1 promoted cell growth through suppressing miR-205 and promoting SMAD4 expression

**Figure 6:**
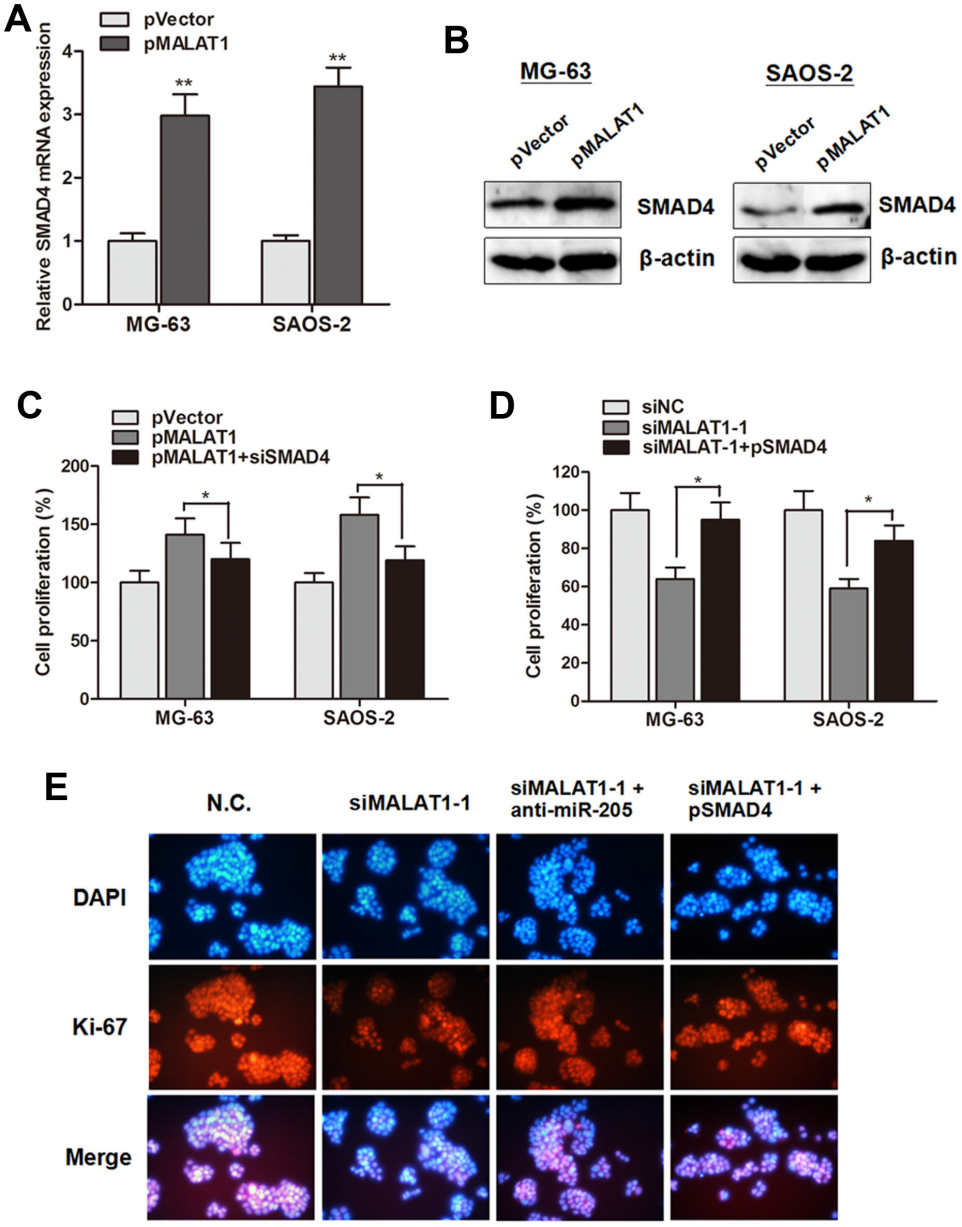
MALAT1 promoted cell growth through suppressing miR-205 and promoting SMAD4 expression **(A-B)** SMAD4 mRNA (A) and protein (B) expression level were significantly up-regulated by overexpression of MALAT1 in MG-63 and SAOS-2 cells; **(C)** CCK8 assay showed that the enhanced cell growth induced by pMALAT1 was abrogated by siSMAD4 in MG-63 and SAOS-2 cells; **(D)** Overexpression of SMAD4 partially reversed the suppressed cell viability caused by siMALAT1-1 in both cell lines; **(E)** Immunofluorescence assay showed that inhibition of MALAT1 significantly suppressed Ki-67 expression level. However, anti-miR-205 or pSMAD4 partially rescued the siMALAT1-1 induced suppression of Ki-67 expression in MG-63 cells. *P<0.05, ^**^P<0.01.

The above data revealed that MALAT1 promoted cell proliferation through suppressing miR-205, and miR-205 suppressed cell growth through targeting SMAD4 in osteosarcoma cells. Thus, we sought to determine whether the cell proliferation effect induced by MALAT1 is through silencing miR-205 and activating SMAD4 function. As shown in Figure 6A and 6B, the mRNA or protein level of SMAD4 was dramatically increased when MALAT1 was up-regulated by pMALAT1 in MG-63 and SAOS-2 cells. Additionally, CCK8 assay showed that the enhanced cell growth induced by pMALAT1 was abrogated by siSMAD4 in MG-63 and SAOS-2 cells (Figure 6C); on the other hand, overexpression of SMAD4 partially reversed the suppressed cell viability caused by Lv-shMALAT1 in both cell lines (Figure 6D). More importantly, the cell proliferation marker Ki-67 was detected by immunofluorescence. Inhibition of MALAT1 significantly suppressed Ki-67 expression level. However, anti-miR-205 or pSMAD4 partially rescued the siMALAT1-1 induced suppression of Ki-67 expression in MG-63 cells (Figure 6E). Collectively, we demonstrated that MALAT1 promotes cell proliferation through suppressing miR-205 and promoting SMAD4 expression in osteosarcoma.

## DISCUSSION

Recent advances in the non-protein coding part of human genome analysis have discovered extensive transcription of large RNA transcripts that lack coding protein function, termed non-coding RNA [18, 19]. It is becoming evident that lncRNAs may be an important class of pervasive genes involved in carcinogenesis [3, 20]. Currently, tumor fast growth and pulmonary metastasis are major reasons of death for patients with osteosarcoma, revealing that effective prognostic factor and therapeutic target could help improve treatment strategies to overcome metastatic osteosarcoma. The aim of this study is to investigate the clinical experimental function of MALAT1 in human osteosarcoma and further investigate the potential regulatory mechanism by which MALAT1 participate in osteosarcoma progression. The present study demonstrated that MALAT1 was significantly up-regulated and predicted poor outcome in osteosarcoma. More importantly, we further uncovered that MALAT1 mediated cell proliferation and cell cycle arrest through a miR-205-SMAD4 signaling pathway in osteosarcoma cells.

MALAT1 is one of the first cancer-associated lncRNAs. Its level is upregulated in cancer tissues and its dysregulation is discovered as a marker for metastasis development in early stages of lung adenocarcinoma and other cancers [21]. High MALAT1 expression was also associated with high stage, metastasis and shorter overall survival after radical nephrectomy and chemotherapy in cancer patients [22–24]. For osteosarcoma, previous reports revealed that MALAT1 was also upregulated in primary tissues and cell lines, and high MALAT1 expression level confers a poor prognosis [25, 26]. More importantly, one study demonstrated that high MALAT1 was associated with poor response to chemotherapy in osteosarcoma patients. However, this conclusion does not retain statistical significance in a multivariate analysis, which may be due to the small sample size [25]. In this study, we identified the up-regulation of MALAT1 in primary osteosarcoma tissues or cell lines and further explored its diagnostic and prognostic value. We found that MALAT1 had relatively high diagnostic value and predicted poor survival rate in osteosarcoma patients.

After having validated the up-regulation of MALAT1, we then investigated the underlying regulatory mechanism that may account for the clinical findings. The interaction between lncRNAs and miRNAs has been reported to play important roles in tumorigenesis, enhanced proliferation, and drug resistance in various malignancies [27, 28]. Li et al found that MALAT1 promote colorectal cancer progression and chemoresistance through interacting with miR-218 [29]. Han et al found that miR-125b suppressed bladder cancer cell carcinoma through influencing the function of MALAT1 [30]. By using the online bioinformatics tool *miRcode* (http://www.mircode.org/mircode), we identified several binding target of miR-205 on the MALAT1 gene sequences. Spearman correlation assay showed that MALAT1 negatively correlated with miR-205 expression in osteosarcoma tissues. More importantly, the gain and loss function assay indicated that MALAT1 can suppress the expression of miR-205, and this interaction has a reciprocal effect.

Human miR-205 was first predicted by computational approaches, based on its high conservation with mouse and Fugu rubripes [31], and its homologs have been discovered among several species. However, the role of miR-205 in osteosarcoma progression is not well known. One study showed that miR-205 associated with the malignant status and poor prognosis in different solid tumors [32, 33]. On the other hand, various reports revealed that miR-205 was a tumor suppressor gene in breast cancer and lung cancer cells by suppressing cell proliferation and promoting apoptosis [34–36]. Thus, this contradictory conclusion indicates that more research is needed to investigate the role of miR-205 in osteosarcoma. For osteosarcoma, miR-205 was also widely accepted as a tumor suppressor gene through suppressing proliferation, migration and invasion [12]. This is consistent with our conclusion in view that we also validated the down-regulation of miR-205. Previously, Wang et al demonstrated that miR-205 suppressed MG-63 cell proliferation and invasive capacity by targeting VEGFA [37]; Yang et al found that miR-205 functioned as an antioncogene by targeting TGF-α [38], while Zhang et al found that miR-205 inhibited osteosarcoma progression through targeting RUNX2 [39]. These observations suggest that miR-205 may be a tumor suppressor, but there are no agreements on how miR-205 exerts its function.

By using the miRNA target predicting databases, we identified SMAD4 as a potential target of miR-205. SMAD4 is a key transducer of transforming growth factor-β (TGF-β) superfamily signaling that is located in chromosome 18q21 and regulates cell proliferation, differentiation, and apoptosis [40]. Animal studies have shown that SMAD4 inactivation is involved in the malignant transformation of gastrointestinal adenomas [41] and a reduction in SMAD4 mRNA levels has been observed during tumor progression [42]. Additionally, SMAD4 has been identified as functional targets of various miRNAs including miR-483, miR-224 and miR-20a-5p [43]. Thus, we focus on the interaction between miR-205 and SMAD4. We found that miR-205 was negatively correlated with SMAD4 expression in osteosarcoma tissues. More importantly, overexpression of miR-205 significantly suppressed SMAD4 expression level in osteosarcoma cells, and Dual-Luciferase reporter assay further indicated a direct binding of miR-205 on SMAD4 3’UTR region. Subsequently, cell gain and loss function assay showed that miR-205 suppressed cell viability by targeting SMAD4, suggesting that miR-205 serves as a tumor suppressor mainly through regulating the function of SMAD4.

Finally, we sought to establish the regulatory pathway of MALAT1-miR-205-SMAD4 in osteosarcoma. SMAD4 was suppressed by MALAT1 inhibitor while promoted by MALAT1 plasmid. More importantly, targeted silencing of SMAD4 could interfere the effect of MALAT1 on cell proliferation. In conclusion, our integrated approach demonstrated that MALAT1 was up-regulated and conferred a poor prognosis in osteosarcoma patients. MALAT1 mediated cell proliferation and cell cycle arrest through suppressing miR-205 and promoting SMAD4 expression. Thus, lncRNA MALAT1 may be a potential prognostic and therapeutic target in osteosarcoma. Suppression of MALAT1 could be a future direction to promote the clinical outcome of osteosarcoma patients.

## MATERIALS AND METHODS

### Clinical samples

Sixty-four cancer tissues and paired adjacent noncancerous tissues (male/female: 40/24, range of age: 17-45) from primary osteosarcoma patients were collected at The Second Hospital of Shandong University between 2010 and 2012. All the patients were pathologically confirmed and the tissues were collected immediately after they were obtained during the surgical operation, and then stored at -80°C to prevent RNA loss. They were classified according to the WHO criteria and staged according to the tumor-node-metastasis (TNM) classification. Written informed consent was obtained from all patients according to the guidelines approved by the Ethics Committee of The Second Hospital of Shandong University.

### Cell culture

Human osteosarcoma cell lines MG-63, SAOS-2, U2OS, SW1353 and one osteoblastic cell line (hFOB) were obtained from the Type Culture Collection of the Chinese Academy of Sciences (Shanghai, China). All osteosarcoma cell lines were maintained in Dulbecco’s Modified Eagle’s Medium (DMEM) medium (Invitrogen, Carlsbad, CA, USA) containing 10% fetal bovine serum (FBS) (Sigma-Aldrich, St. Louis, MO, USA), 100 U/ml penicillin and 100 g/ml streptomycin (Life Technologies, Grand Island, NY, USA) at 37 °C in 5% CO_2_ and 95% air. Osteoblastic hFOB cells were grown in DMEM/F12 1:1 medium with 10% FBS, 2.5 mM L-glutamine and 0.3 mg/ml G418 at 37 °C in 5% CO_2_ and 95% air. The cell lines passed the DNA profiling test (STR).

### RNA oligoribonucleotides and cell transfection

The small interfering RNAs (siRNAs) that specifically target human lncRNA MALAT1, miR-205 and SMAD4 mRNA were designated as siMALAT1, anti-miR-205 and siSMAD4, respectively. The MALAT1 overexpression plasmid (pMALAT1) was purchased from Addgene. The coding sequence of SMAD4 was amplified and then cloned into PCDNA3.1 vector, and was named as pSMAD4. The lentiviras vector containing MALAT1 ShRNA plasmid (Lv-ShMALAT1) was amplified and cloned (Genechem corporation, Shanghai, China). The negative control duplex (NC) for both miRNA mimics and siRNA, as well as the single standard negative control RNA for miRNA inhibitors (anti-NC), was not homologous to any human genome sequences. All RNA oligoribonucleotides were purchased from RiboBio (Guangzhou, China). The transfection of RNA oligoribonucleotides and plasmid was performed by using Lipofectamine 2000 (Invitrogen).

### Dual-luciferase reporter assay

The putative miR-205 binding sites in the SMAD4 3’-UTR was predicted by TargetScan and miRanda. Dual-luciferase reporter assay was performed using pmiR-REPORT™ vectors (RiboBio) containing wild-type SMAD4 3’-UTR sequences or mutant SMAD4 3’-UTR sequences. Cells (1×10^5^) were transiently transfected with miR-205 mimics or negative control together with wild-type SMAD4 3’-UTR vector or mutant type SMAD4 3’-UTR vector in a 24-well plate. Cells were harvested 48 h after transfection, and luciferase activity was analysed by the Dual-luciferase Reporter Assay Kit (Promega, Madison, WI, USA) according to the manufacturer’s instructions.

### Quantitative real-time PCR (RT-qPCR)

Total RNA was isolated from primary osteosarcoma tissues or osteosarcoma cell lines using TRIzol reagent (Invitrogen). And then, the cDNA was synthesized from 200 ng extracted total RNA using the PrimeScript RT reagent Kit (Takara Bio Company, Shiga, Japan) and amplified by RT-qPCR with an SYBR Green Kit (Takara Bio Company) on an ABI PRISM 7500 Sequence Detection System (Applied Biosystems, Foster City, CA, USA) with the housekeeping gene GAPDH as an internal control. The 2^-ΔΔCt^ method was used to determine the relative quantification of gene expression levels. All the premier sequences were synthesized by RiboBio, and the premier sequences were as follows: MALAT1 (Forward): GGGTGTTTACGTAGACCAGAACC, (Reverse): CTTCCAAAAGCCTTCTGCCTTAG; SMAD4 (Forward): CAGCTATGCCAGAAGCCAGA, (Reverse): GAACTCCTGGGACTTTCAACTGAC; GAPDH (Forward): GCACCGTCAAGGCTGAGAAC, (Reverse): ATGGTGGTGAAGACGCCAGT. Each experiment was performed in triplicate.

### Cell proliferation assay

Cell proliferation was quantified by using the Cell Counting Kit-8 (CCK-8, Beyotime Corporation, Shanghai, China). Briefly, 100 μl of cells from the different transfection groups were seeded onto a 96-well plate at a concentration of 2000 cells per well and were incubated at 37 °C. At different time point, the optical density was measured at 450 nm using a microtiter plate reader, and the rate of cell survival was expressed as the absorbance. The results represent the mean of three replicates under the same conditions.

### Cell cycle analysis

After transfection, cells were washed in PBS and fixed in 70% ethanol at 4°C for 2 hours. DNA staining was done with 10 mg propidium iodide/mL PBS and 2.5 Ag DNase-free RNase (Roche Diagnostics)/mL PBS for at least 30 minutes before flow cytometry in a Coulter EPICS XL flow cytometer (Beckman Coulter, Fullerton, CA, USA). Cell cycle profiles were generated from flow cytometry analysis with Modifit software (BD Biosciences, Franklin, NJ, USA).

### Immunohistochemistry

Paraffin-embedded, formalin-fixed tissues were immunostained for SMAD4 using a rabbit anti-SMAD4 primary antibody (9515S, Cell Signaling Technology, Beverly, MA, USA) at 1:1000 dilution. Five-micron tissue slides from tumor tissue were de-paraffinized using xylene. Heat-mediated antigen retrieval was performed using citrate buffer (BioGenex Laboratories, San Ramon, CA). Antibody staining was visualized with DAB (Sigma, D-5637) and hematoxylin counterstain. Semi-quantitative IHC detection was used to determine the SMAD4 protein levels. Using the H-score method, we multiplied the percentage score by the staining intensity score. Immunohistochemical scoring was performed without prior knowledge of the clinical response.

### Immunofluorescence analysis

MG-63 cells were grown to 40% to 50% confluence and then transfected with 100 nM of siMALAT1-1, miR-205 mimics or si-TS. After 48 hours of incubation, the cells were fixed with 4% paraformaldehyde and permeabilized in 0.2% Triton X-100 (Sigma-Aldrich) for 20 minutes. The cells were then blocked with 10% goat serum in PBS for 1 h. Cells were incubated with primary anti-Ki-67 (Cell Signaling Technology) overnight at 4°C and then incubated with the appropriate rhodamine-conjugated secondary antibody for 1 h. The cells were then washed and incubated with DAPI (Invitrogen) for nuclear staining. The slides were visualized for immunofluorescence with a laser scanning Olympus microscope.

### Western blot and antibodies

The primary antibodies used for western blotting were rabbit anti-human SMAD4 antibody (1:1000; Cell Signaling Technology) and rabbit anti-human β-actin antibody (1:1000; Cell Signaling Technology). Horseradish peroxidase-conjugated (HRP) anti-rabbit antibodies (1:5000; Santa Cruz Biotechnology, Santa Cruz, CA, USA) were used as the secondary antibodies. A total of 25 μg protein from each sample was separated on 10% Bis-Tris polyacrylamide gel through electrophoresis and then blotted onto polyvinylidene fluoride (PVDF) membranes (GE Healthcare, Piscataway, NJ, USA). Then, the membrane was blocked with 5% (5 g/100 mL) nonfat dry milk (Bio-Rad, CA, USA) in tri-buffered saline plus Tween (TBS-T) buffer for 2 h. Blots were immunostained with primary antibody at 4°C overnight and with secondary antibody at room temperature for 1 h. Immunoblots were visualised by using Immobilon™ Western Chemiluminescent HRP Substrate (Millipore, Bedford, MA, USA). Protein levels were normalized to β-actin.

### Statistical analysis

The differences of lncRNAs or miRNAs expression level between different groups were analyzed by the Mann-Whitney U-test or Kruskal-Wallis test. Correlation analyses were carried out using Spearman’s rank correlation method. Receiver operating characteristic (ROC) curves were established to discriminate osteosarcoma responding patients from non-responding patients. AUC was used as an accuracy index for evaluating the predictive performance of MALAT1. A log-rank test was used to analyze the statistical differences in survival as deduced from Kaplan-Meier curves. Cox proportional-hazard regression analysis was performed to calculate HR and 95% CI for each covariable. All differences were regarded as statistically significant when *P*<0.05. MedCalc 9.3.9.0 (MedCalc, Mariakerke, Belgium) was used for ROC analysis, and other statistical analyses were performed with GraphPad Prism 5.01 (GraphPad Software, La Jolla, CA, USA).
